# Exposure to High-Intensity Light Systemically Induces Micro-Transcriptomic Changes in *Arabidopsis thaliana* Roots

**DOI:** 10.3390/ijms20205131

**Published:** 2019-10-16

**Authors:** Barczak-Brzyżek Anna, Brzyżek Grzegorz, Koter Marek, Gawroński Piotr, Filipecki Marcin

**Affiliations:** 1 Department of Plant Genetics, Breeding and Biotechnology, Institute of Biology, Warsaw University of Life Sciences–SGGW, 02-776 Warszawa, Poland; barczak.annak@gmail.com (B.-B.A.); marek_koter@sggw.pl (K.M.); 2 Institute of Biochemistry and Biophysics Polish Academy of Sciences, 02-106 Warszawa, Poland; grzesiekbrzyzek@gmail.com

**Keywords:** miRNA, high light, abiotic stress, systemic response, roots

## Abstract

In full sunlight, plants often experience a light intensity exceeding their photosynthetic capacity and causing the activation of a set of photoprotective mechanisms. Numerous reports have explained, on the molecular level, how plants cope with light stress locally in photosynthesizing leaves; however, the response of below-ground organs to above-ground perceived light stress is still largely unknown. Since small RNAs are potent integrators of multiple processes including stress responses, here, we focus on changes in the expression of root miRNAs upon high-intensity-light (HL) stress. To achieve this, we used *Arabidopsis thaliana* plants growing in hydroponic conditions. The expression of several genes that are known as markers of redox changes was examined over time, with the results showing that typical HL stress signals spread to the below-ground organs. Additionally, micro-transcriptomic analysis of systemically stressed roots revealed a relatively limited reaction, with only 17 up-regulated and five down-regulated miRNAs. The differential expression of candidates was confirmed by RT-qPCR. Interestingly, the detected differences in miRNA abundance disappeared when the roots were separated from the shoots before HL treatment. Thus, our results show that the light stress signal is induced in rosettes and travels through the plant to affect root miRNA levels. Although the mechanism of this regulation is unknown, the engagement of miRNA may create a regulatory platform orchestrating adaptive responses to various simultaneous stresses. Consequently, further research on systemically HL-regulated miRNAs and their respective targets has the potential to identify attractive sequences for engineering stress tolerance in plants.

## 1. Introduction 

The simplistic model of plant roots taking up water and nutrients that are essential for plant growth and receiving from shoots sugars and auxins which drive root development is much more complicated than an availability–growth relationship. In recent years, knowledge about the role of roots as a component of the plant signaling network integrating environmental cues has greatly expanded, revealing roots’ central role in optimizing plant nutrient demand in response to shoot-derived stress signals and changes in photosynthesis capacity [1,2]. The rate of carbon assimilation reflects the condition of the photosynthetic apparatus and depends on light availability, which varies according to season, diurnal rhythm, and canopy structure, in terms of both light intensity and spectrum. In terms of light intensity, two extreme situations can occur: (1) light deficiency and (2) excess light (EL) caused by high light intensity (HL). Because such HL incidents may lead to photoinhibition, photoprotective mechanisms are triggered to avoid or dissipate the excess of light energy. These mechanisms include ultrastructural adaptations (e.g., chloroplast movement and thylakoid proteins arrangement), physical energy dissipation (e.g., by heat and chlorophyll fluorescence), and a number of biochemical processes such as photochemical and non-photochemical quenching, chlororespiration, photorespiration, production of antioxidant enzymes (e.g., APX, SOD) as well as carotenoids, tocopherols, or small antioxidant molecules (e.g., ascorbate and glutathione) [3,4,5,6,7,8].

Given the substantial role of light in the regulation of plant developmental processes and stress response, it is not surprising that plants have an extremely sensitive light-sensing system, with photoreceptors dedicated to different wavelengths of light [1,9]. These photoreceptors include cryptochromes (CRYs) and phototropins (PHOT), which detect UV-A and blue light, phytochromes (PHYs), which detect red (R) and far-red (FR) light, and UV-B resistance locus 8 (UVR8), which detects UV-B light [1,9,10]. Since light may penetrate only several centimeters under the ground surface, for many years it was a puzzle how plant roots perceive light [9]. Although root anatomy, morphology, and physiology were observed to be regulated by light, the majority of the changes were attributed to changes in sugar availability due to fluctuations in light intensity and thus in photosynthesis. However, a growing body of evidence indicates that many aspects of these light systemic responses are part of a more complex signaling network. This was proven by studies that documented that photoreceptors occur all over the plant body, including in dark-grown roots. Additionally, in recent years, physical and genetic approaches have suggested that photoreceptors expressed in roots directly sense light [11,12]. For example in *Arabidopsis thaliana* plants, it was shown that a signal triggered by light was conducted through the stems to the roots, where photoactivated phytochrome B (phyB) triggered the expression of the transcription factor elongated hypocotyl 5 (HY5), and that there was a consequent accumulation of HY5 protein which resulted in the promotion of root growth in response to light [11,12]. Another breakthrough finding concerning light-regulated shoot-to-root signaling was presented by Chen et al. [13], who demonstrated that HY5 itself is a mobile signal which travels from rosettes to the underground part of *A. thaliana* plants. In the above-ground parts, HY5 underpins shoot growth with C assimilation, whereas in roots, it stimulates growth-enhancing N uptake by the up-regulation of NRT2.1, which encodes a major root NO_3_^−^ transporter. These observations strongly support the idea that light is essential for the plant signaling network which modulates nutrient uptake and demand at the whole-plant level under different environmental conditions [14]. Such a long-distance signaling network is crucial for relaying information about the local environment and may involve different molecular components. One of the common components of such signaling, a possible stress integrator, is microRNA (miRNA). Its ability to travel through the plant has been well documented in the case of phosphate (P) homeostasis regulation, where *Arabidopsis* miR399 appears to be an important regulator during P starvation [15,16,17,18]. Furthermore, miRNAs have been proven to play a role in many other nutrient-related responses [19,20,21] as well as in responses to other abiotic [22,23] and biotic stresses [24,25]. However, in most of these studies, long-distance miRNA transport was not proven. Systemic changes in miRNA expression in response to stress factors may engage other signaling molecules, such as mobile RNAs or peptides, molecules of a different nature, such as reactive oxygen species (ROS) or phytohormones, as well as calcium waves or electrical signaling [26,27,28,29]. 

Although miRNAs are major regulators of gene expression, their role in light stress signaling is not well understood, especially in the context of root response [30]. This is rather surprising, given that about 20% of genes in *Arabidopsis* are regarded as light-responsive [30,31,32,33]. Further expanding the knowledge about the role of miRNAs in HL-stressed plants is extremely interesting in the context of the recent study of Petrillo et al. [32]. They proved that splicing—one of the mechanisms by which gene expression is regulated by light—occurs not only in shoots but also in roots and observed changes that are regulated by redox signals derived from chloroplasts. Since splicing may influence miRNA expression, the question arises: Can HL stress directly or indirectly regulate the expression of root miRNAs, and what is the nature of the shoot-derived stress signal? To address this question, we analyzed HL-triggered changes in the expression of miRNAs in roots using a micro-transcriptomic approach followed by RT-qPCR analysis. Direct exposure of roots (separated from shoots before exposure to HL) to HL stress revealed that a stress signal is induced in rosettes and travels through the plant, thereby affecting the expression of miRNAs. The list of potential targets of HL-regulated root miRNAs opens interesting perspectives for engineering stress responses in plants.

## 2. Results

### 2.1. HL-Induced Transcriptional Changes in A. thaliana Roots

**Figure 1:**
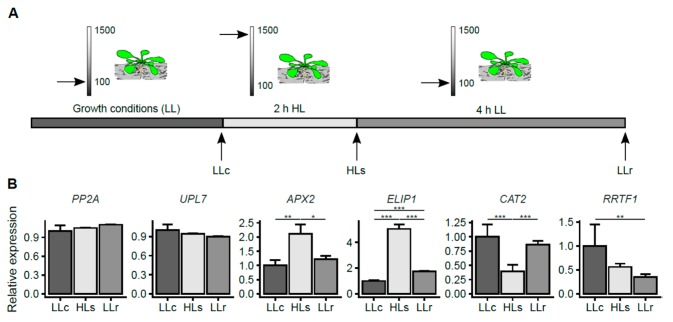
The transcriptional response of stress markers in *Arabidopsis thaliana* roots after the exposure of rosettes to high light intensity (HL). (**A**) Experimental scheme: LLc: roots of plants grown in low-light (LL) control conditions (2 h after the LL day started); HLs: roots of plants exposed to 2 h of HL stress; LLr: roots of plants exposed to 2 h of HL followed by 4 h of recovery in LL conditions. The arrows on the scales indicate the light intensity (µmol photons m^−2^s^−1^). (**B**) Relative expression of the marker genes compared to the genes *PP2A* and *UPL7*. Error bars represent the standard deviation, and asterisks represent significant differences at *p*-values <0.05 (*), <0.01 (**), and <0.001 (***). ANOVA and the HSD Tukey test were applied for statistical analysis. Results from two independent experiments were each pooled from six plants and three technical replicates.

To study the HL response in *A. thaliana* roots, we used plants growing in a hydroponic system, which enables the continuous growth of roots in the dark and minimizes mechanical damage and stress. Plants growing in low-light conditions (LL; 100–120 µmol photons m^−2^ s^−1^) and a short-day photoperiod (Figure 1A) were subjected to two hours of HL stress at an intensity of 1500 µmol photons m^−2^ s^−1^, and the expression of several HL-response-related genes was checked immediately following the stress and after 4 h of recovery, to confirm whether the stress signal spread to dark-grown roots. 

HL response markers were selected on the basis of previous research which showed that an acclimation response was induced within minutes of episodes of HL in stressed tissues and was transmitted to HL-unexposed leaves of the same plant, thereby initiating an HL systemic acquired acclimation response (HL-SAA) [34,35]. HL-SAA is mainly regulated by retrograde signals derived from chloroplasts, such as changes in the plastoquinone (PQ) redox status pool, ROS production, or other retrograde signaling pathways [36,37,38,39,40,41,42,43]. The subsequent activation of photoprotective mechanisms is orchestrated by the transcriptional activation of many HL-, ROS-, hormone-, pathogenesis-, and drought-related genes [44,45,46]. 

In this study, we analyzed the root transcript level of two enzymes that are directly involved in ROS detoxification, namely, ascorbate peroxidase 2 (*APX2* gene) and catalase 2 (*CAT2* gene), as well as the transcript level of redox responsive transcription factor 1 (*RRTF1* gene) and early light inducible protein 1 (*ELIP1* gene), which have both been described as markers of HL-SAA [34,38]. Our analysis covered three consecutive time points: (1) before the HL stress (low-light control, LLc); (2) immediately after the HL stress episode (HLs); and (3) four hours of recovery in LL after HL stress (LLr) (Figure 1A,B). The transcript levels of *APX2* and *ELIP1* increased significantly within two hours of HL stress and decreased after the subsequent four hours of recovery. The transcript level of *APX2* quickly reverted back to its LLc level after the HL episode; in contrast, after four hours of recovery, the transcript level of *ELIP1* was still significantly up-regulated compared to the control. The transcript level of *CAT2* decreased immediately after the HL episode, however, it returned to the control level after four hours in LL conditions. Meanwhile, the expression of *RRTF1* decreased and remained low after four hours in low-light conditions (Figure 1B). These transcriptional changes in the expression of light-stress marker genes confirmed the induction of a stress response in roots kept in darkness.

### 2.2. HL-Triggered Systemic Changes in the Expression of miRNAs 

**Figure 2:**
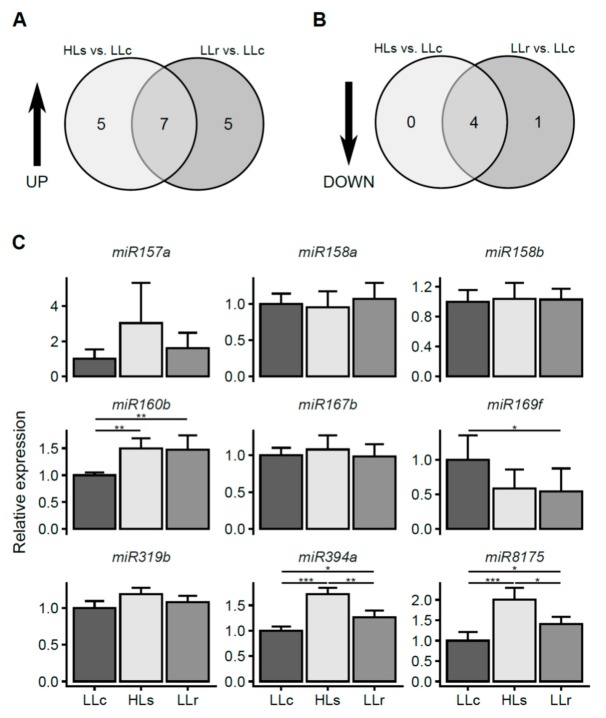
Micro-transcriptomic changes in *A. thaliana* roots induced by HL treatment of rosettes (**A**) Venn diagram representing the number of miRNAs that were up-regulated after HL treatment compared to control roots. (**B**) Venn diagram representing the number of miRNAs that were down-regulated after HL treatment compared to control roots. (**C**) Validation of selected micro-transcriptomic changes in miRNA expression, relative to the expression of two references, *snoRNA85* and *snoRNA101*, using the RT-qPCR method. Error bars represent the standard deviation, and asterisks represent significant differences at *p*-values <0.05 (*), <0.01 (**), and <0.001 (***). ANOVA and the HSD Tukey test were applied for statistical analysis. Results are from three independent experiments each pooled from six plants and three technical replicates.

After verifying that the stress signal was transferred from shoots to dark-grown roots and resulted in changes in the expression of HL marker genes, we performed micro-transcriptomic analysis (see Supplementary Table S1). The obtained data were filtered using the cutoff fold change (FC) ≤ 0.65 and FC ≥ 1.5, which allowed us to select a relatively limited set of 17 up-regulated and 5 down-regulated miRNAs (Figure 2A,B; Supplementary Table S1). Among the up-regulated miRNAs, the expression activation of five miRNAs was restricted to one time point, while the expression activation of seven miRNAs was induced in both treatments (immediately after the HL stress and after four hours of recovery, respectively). Of the five miRNAs whose expression was down-regulated in response to the HL episode, four were commonly down-regulated in the HLs and LLr conditions, and one was down-regulated exclusively in the LLr condition. No miRNAs were down-regulated only in the HLs condition. The micro-transcriptomic screening was further validated using two RT-qPCR methods. The first one, which was performed according to Androvic et al. [47], used two-tailed RT target-specific primers consisting of two hemiprobes, whereas the second one was based on a universal RT reaction (for details, see the Materials and Methods section and Supplementary Table S2). We observed statistically significant changes in the micro-transcriptomic data for three up-regulated miRNAs (miR160b, miR394a, and miR8175) and one down-regulated miRNA (miR169f). Changes were also observed in the induction of miR157a, however, they were not statistically significant (Figure 2C).

### 2.3. Stress Signal is Induced in Rosettes

**Figure 3:**
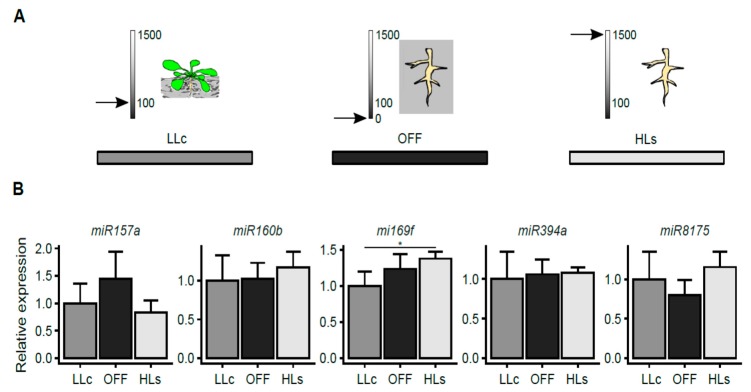
Verification of the stress signal source. (**A**) Experimental scheme: LLc: control roots (collected from plants grown in LL for 2 h); OFF: roots dissected from shoots as in LLc and kept in the dark for 2 h; HLs: roots dissected from shoots as in LLc and exposed to HL for 2 h. The arrows on the scales indicate light intensity (µmol photons m^−2^s^−1^). (**B**) Expression level of miRNAs relative to two references, *snoRNA85* and *snoRNA101*. Error bars represent the standard deviation, and asterisks represent significant differences at *p*-values < 0.05 (*). ANOVA and the HSD Tukey test were applied for statistical analysis. Results are from three independent experiments each pooled from six plants and three technical replicates.

Next, we investigated whether the light stress signal which caused the changes in miRNA expression in roots could originate from rosettes only or whether it could also be generated in HL-exposed roots. To test this, the roots were separated from the shoots and subsequently exposed to 2 h of HL (Figure 3A). Additionally, taking into consideration the possible effect of wounding on the level of expression of miRNA, roots dissected from shoots that were kept in darkness were also included in the analysis (OFF; see Figure 3A). For all HL stress-regulated miRNAs, we were unable to induce similar local changes in HL-exposed roots (Figure 3B). Only in the case of miR169f, we observed a slight HL induction which was opposite to the effect observed in the shoot–root experiment and similar to the trend of the wounding reaction (Figure 3B). Interestingly, in two cases, namely, miR157a and miR8175, the local HL stress seemed to abolish the slight effect of mechanical root detachment. The above data suggest that the main light stress signals were generated in rosettes. 

### 2.4. Prediction of Potential Targets

Since the miRNAs are able to specifically suppress individual protein-coding genes, the identification of mRNA targets is the first and most important step in interpreting miRNAs engagement in plant growth, development, or stress response. Therefore, we used the psRNATarget software to predict potential targets for the differentially expressed miRNAs (Table 1, Supplementary Table S3) [48].

Only one candidate, miR160b, targets transcription factors, namely, auxin response factors (ARFs), which have been previously proven to be regulated by miRNA. Another one, miR169f-3p, targets an important enzyme in phosphoinositide signaling. Interestingly, two other miRNAs, miR394a and miR8175, may target transcripts of nuclear-encoded, important players in chloroplast biogenesis and functioning. These are promising candidates for future research, which should be extensively functionally analyzed. 

## 3. Discussion 

Above-ground and underground parts of plants play different roles, which must be synchronized to ensure optimal growth and development under various environmental cues [10]. Since light is both extremely variable and the most powerful factor which influences plant performance, plants should possess robust and precise molecular regulatory mechanisms that are deeply integrated into any physiological process. Indeed, light plays its role not only by regulating photosynthesis but also by possibly cross-talking with nutrient acquisition and the regulation of the stress response both locally and systemically [14,35,54]. 

In this study, we analyzed the high-light stress response of *A. thaliana* roots grown in darkness. The root response was confirmed by monitoring the transcripts typically induced in shoots upon oxidative stress: *APX2* and *CAT2*. *APX2* reacted by a strong induction and recovered to its initial level after 4 h, which is in accordance with its role in systemic acclimation to stress [34]. The *CAT2* transcript was down-regulated just after the HL treatment but also recovered within 4h of LL. This reaction is different from the induction observed in rosette leaves, indicating that oxidative stress signal transduction reaches the roots but is not related to elevated levels of H_2_O_2_ [55]. The observed *RRTF1* gene expression supports this notion, showing transcriptional down-regulation in the roots immediately after HL and maintaining a low level even 4 h after stress exposure. This is in line with earlier reports by Matsuo et al. [56]. ELIP1 is a nuclear-encoded, chlorophyll a⁄b-binding-related protein, localized in the chloroplast thylakoid membranes, which is differentially transcribed in response to light stress in the leaves, with a possible photoprotective role, preventing photo-oxidation or dissipating excess energy [57]. Its role, however, can be wider including its participation in the phytochrome signaling pathway leading to seed germination in tomato and *Arabidopsis* [58,59]. In this report, we observed a strong *ELIP1* induction in shaded roots of *Arabidopsis* plants treated with HL, indicating that similarly to other above-described genes, there are stress signals generated in the shoots that move to the underground plant parts. This observation leads also to the question of what the *ELIP1* role could be in roots that do not contain chlorophyll and have a number of plastids much lower than in leaf mesophyll. Despite the fact that this is a fascinating question, in the present report, we will focus on regulatory mechanisms evoked by aboveground HL episodes influencing shaded root functioning. 

An interesting mediator of such information exchange is miRNA, which is a sequence-specific and potent post-transcriptional, negative regulator of gene expression. Several reports have described the complexity of how light influences miRNA expression following a variety of light treatments [30,31,60]. Despite the crucial role of roots in the optimization of plant growth by the spatial and temporal adjustment of nutrient demand to photosynthetic capacity under various environmental constraints, their significance is often overlooked. 

Using micro-transcriptomic analysis, we were able to select candidates for subsequent validation by RT-qPCR methods, which confirmed the significant up-regulation of miR160b, miR394a, and miR8175, while down-regulation was shown only for miR169f. Since previous reports described that alternative splicing (AS) is regulated by light in both shoots and roots and proved that the observed changes in splice variants proportions diminished when roots were dissected from rosettes before exposition to light [32], our further study was directed towards checking if the same is true for the regulation of miRNA expression. By exposing dissected roots to HL, we proved our assumption that the stress signal was generated in rosettes. Although the effect of light on AS required functional chloroplasts and was initiated by changes in the PQ redox status, the nature of the signaling molecules that travel through plants is still unknown [32]. Moreover, there may be crosstalk between AS and miRNA-mediated post-transcriptional gene regulation, which makes the study of the effects of light on these two important gene regulatory mechanisms extremely interesting [61]. For example, in *Physcomitrella patens*, it was shown that most of the factors involved in miRNA processing undergo AS [61,62]. Additionally, it was recently found that light may regulate miRNA processing by changing the phosphorylation state of hyponastic leaves 1 (HYL1), an important player in miRNA maturation. An extended period of light deprivation led to the degradation of HYL1, while the restoration of light resulted in the dephosphorylation of HYL1 protein and switched on miRNA biogenesis [63]. Since light may regulate many steps of miRNA biogenesis, including transcription, splicing, stabilization, and degradation, much more work should be performed to explore these areas of plant biology [64].

An obvious complement to the research on pathways involving miRNAs is the identification of their targets. Such work could open the way to the application of genetic engineering to change agronomic traits. One example in which such a strategy was successfully applied is the manipulation of chloroplastic superoxide dismutase (CSD2) as a target of *miR398*, which improved plant tolerance to HL, heavy metals, and other oxidative stresses [65]. 

Among miRNAs in HL-regulated *Arabidopsis* root, ath-miR160b is known to regulate the ARF family genes *ARF10*, *ARF16*, and *ARF17*. *ARF10* and *ARF16*, although functionally redundant, have been shown to be involved in the determination of *Arabidopsis* root architecture. The miR160-dependent down-regulation of *ARF10* and *ARF16* led to a reduction in main-root gravitropism and main-root length and to an increase in the number of lateral roots [66]. *ARF17* has been shown to be a negative regulator of acyl-acid-amido synthetases (GH3s) involved in the formation of inactive jasmonic acid (JA) conjugates, leading to a low level of active JA–Ile, which negatively modulates the adventitious rooting process through the activation of the COI1 signaling pathway [67]. Thus, the observed up-regulation of miR160 may generate a signal for root system expansion via the inhibition of all potential ARF targets. Moreover, the specificity of JA–Ile partially overlaps with that of JA, a hormone involved in herbivore resistance; however, due to the lower activity of JA–Ile and the availability of mainly shoot-derived data [68], the possible role of miR160 in managing trade-offs between stress tolerance and growth and reproduction is unclear and requires more research. 

The miRNA miR169f-3p is the only confirmed candidate which was significantly down-regulated in roots upon HL stress and remained suppressed after four hours of recovery in low light conditions. Its most significant putative target is a probable phosphoinositide phosphatase, SAC9, which may terminate stress-induced signaling via phosphoinositides (PIs), signaling molecules that regulate cellular events including vesicle targeting and interactions between membrane and cytoskeleton. The *sac9* mutant of *Arabidopsis* accumulates elevated levels of phosphoinositides and produces characteristics of a constitutive stress response, including dwarfism, closed stomata, and anthocyanin accumulation. Moreover, the *sac9* mutant overexpresses stress-induced genes and over-accumulates reactive oxygen species [51]. Since miR169f-3p down-regulation should enhance *SAC9* expression and consequently should have an adverse effect, this might suggest that it plays a different role in roots.

In the case of two other miRNA targets, research suggests their possible role in regulating nuclear genes coding for proteins involved in basic processes in chloroplasts, namely, translation (miR394a/*HCF109* [52]) and plastid division (miR8175/*FtsZ2-1* [53]). We speculate that plastids, besides the relatively low abundance in root cells, may nevertheless function there as specific stress hubs [69,70], which can explain the existence of systemically induced and miRNA-dependent regulatory modules. Engagement of *Arabidopsis* root leucoplasts by the stress response was also postulated by Itoh and Fujiwara [71]. Alternatively, the observed miRNA dynamics might be a residual effect of processes observed in the above-ground parts of the plant, suggesting an interesting involvement of miRNA in retrograde regulation [72] as well as miRNA shoot-to-root movement [73].

The present study contributes to the understanding of plant function in changeable environments regarding the potential of the miRNA-mediated machinery in shoot-to-root communication. The results of this study should be useful for the development of stress-tolerant crops, but first, many hypotheses have to be verified experimentally.

## 4. Material and Methods

### 4.1. Plant Material and Growing Conditions

The *A. thaliana* plants used in this study were a Col-0 ecotype (Nottingham *Arabidopsis* Stock Centre ID 76778). Seeds were surface-sterilized using the chlorine gas method according to Lindsey et al. [74]. Seeds were put in PCR tubes placed in a plastic rack in a desiccator and then exposed to approximately 6% Cl_2_ for 3 h. Next, the tubes were placed in laminar flow hood to eliminate the chlorine gas and then sowed and kept for two days at 4 °C to synchronize germination. Before analyses, the *A. thaliana* plants were grown in hydroponic conditions for 4 weeks, on the basis of Conn et al. method [75]. Briefly, sterilized seeds were placed in black Eppendorf tubes filled by half-strength Murashige and Skoog medium in a plastic opaque container with basal nutrient solution (for details see [73]) which was changed once a week in the first 3 weeks and then twice during the fourth week. During the whole experiment, the roots were protected from light. Controlled growth conditions were set to short day (8 h light/16 h dark; 22 °C/20 °C), 70% air humidity, and low light intensity (LL; 100–120 µmol photons m^−2^s^−1^).

### 4.2. High-Light Treatment

HL treatments were performed by the exposure of LL-adapted plants (2 h after the day started) to HL stress for 2 h using LED light sources with a light intensity of 1500 µmol photons m^−2^s^−1^ (Photon Systems Instruments, Brno, Czech Republic).

### 4.3. Root Separation 

The roots of plants growing hydroponically in LL conditions (2 h after the day started) were separated from the shoots using a scalpel and then put into Petri dishes (150 mm diameter) with three layers of laboratory filter paper soaked with Basal Nutrient Solution (medium composition according to [75]). The Petri dishes were kept in the dark (OFF) or exposed to HL for 2 h (HLs).

### 4.4. RNA Isolation 

RNA extraction was performed using a Universal RNA/miRNA purification kit (EUR_X_, Gdańsk, Poland) according to the manufacturer’s instructions. RNA was eluted using 50 µL of RNAse-free water. The RNA concentration was estimated using NanoDrop 1000 (Thermo Fisher Scientific, Wilmington, MA, USA), and RNA integrity was confirmed using an Experion Automated Electrophoresis System (Bio-Rad, Hercules, CA, USA). In all cases, one biological replicate was pooled from six independent plants.

### 4.5. Preparation of Libraries and Micro-Transcriptomic Analysis

The preparation of miRNA libraries was outsourced to Genomed S.A. (Warsaw, Poland). Briefly, libraries were prepared using the NEBNext® Small RNA Library Prep Set for Illumina® (Multiplex Compatible). Sequencing was performed using the Illumina HiSeq 4000 platform (Illumina Inc., San Diego, CA, USA). The results were analyzed according to the pipeline in Supplementary Figure S1. 

### 4.6. Expression of Marker Genes Determined by RT-qPCR

#### 4.6.1. cDNA Synthesis

Reverse transcription was performed using a Quantitect Reverse Transcription kit (Qiagen, Hindel, Germany) according to the manufacturer’s instructions. cDNA was synthesized from 1 µg of total RNA.

#### 4.6.2. Quantitative PCR

Quantitative RT-PCR was performed in triplicate using a Bio-Rad CFX96 Touch TM Real-Time PCR Detection System (Bio-Rad, Hercules, CA, USA) with the primers listed in Supplementary Table S2. Real-time PCR cycling conditions were optimized depending on the primer used in the protocol, and relative expression was calculated relative to the *UPL7* (AT3G53090) and *PP2A* (AT1G13320) genes. Product melting curves were generated following PCR to ensure the purity of the amplification products.

### 4.7. Detection of Mature miRNAs Using Two-Tailed qPCR

#### 4.7.1. cDNA Synthesis

Reverse transcription for miRNAs was performed with a qScript flex cDNA synthesis kit (Quantabio, Beverly, MA, USA) according to Androvic et al. [47] in the total reaction volume of 10 µL. RNA was diluted in TE-LPA buffer (TE buffer with linear polyacrylamide at a final working concentration of 20 µg/mL). The RT reaction mixture contained 10 ng of total RNA, 1 × RT buffer, 0.05 µM RT primer, 1 µL GSP enhancer, and 0.5 µL RT enzyme. RT reactions were incubated in PCR tubes for 45 min at 25 °C and for 5 min at 85 °C and then held at 4 °C.

#### 4.7.2. Quantitative PCR 

qPCR was performed according to Androvic et al. [47] in a total reaction volume of 10 µL containing 1 × SYBR (BiochemDevelopment, Gdańsk, Poland), 0.4 µM forward and reverse primers, and 0.4 ng of cDNA diluted product. The reactions were performed in triplicates and incubated in a CFX 96 Real-Time Detection System (96 well plates; Bio-Rad, Hercules, CA, USA) at 95 °C for 30 s, followed by 45 cycles of 5 s at 95 °C and 15 s at 60 °C. Reaction specificity was assessed by melting curve. The relative expression level was calculated relative to snoRNA85 (NCBI Accession Number AJ505658) and snoRNA101 (NCBI Accession Number AJ505631).

### 4.8. Detection of Mature miRNA Using Mir-X miRNA 

#### 4.8.1. cDNA Synthesis

Reverse transcription was performed using 300 ng RNA and a Mir-X miRNA First-Strand Synthesis Kit (Takara Bio Inc., Kusatsu, Japan). The total volume of the reaction mixture was 10 µL. The reverse transcription was performed at 37 °C for 1 h followed by enzyme inactivation at 85 °C for 5 min.

#### 4.8.2. Quantitative PCR

qPCR was performed in a total reaction volume of 20 µL containing 10 µL SYBR (BiochemDevelopment, Gdańsk, Poland), 4 µL cDNA diluted product (1 ng µL^−1^), and two mixed template-specific primers (10 µM) designed using the miRPrimer software (see Supplementary Table S1). The reactions were performed in triplicates and incubated in a CFX 96 Real-Time Detection System (96 well plates; Bio-Rad, Hercules, CA, USA). Reaction specificity was assessed by melting curve. The relative expression level was calculated relative to snoRNA85 (NCBI Accession Number AJ505658) and snoRNA101 (NCBI Accession Number AJ505631).

### 4.9. Target Transcript Prediction

Target transcripts for the presented miRNAs were predicted using the psRNATarget Database [48]. Search parameters were set to default, except the maximum expectation, which was set to 2.5, and the length for complementarity scoring, which was 19. The *A. thaliana* unigene DFCI Gene Index (AGI; version 15, released on 2010_04_08) library was used.

### 4.10. Statistical Analysis

Statistical analysis was performed with the R software v.2.13.

## Figures and Tables

**Table 1:** Most significant hits from the prediction of mRNA targets for confirmed miRNAs, obtained using the psRNATarget software. More potential targets and search parameters are presented in Supplementary Table S3.

miRNA	Target ID	Target Name	Target Function	References
ath-miR160b	AT2G28350.1	ARF10	Response to auxin signaling	[49,50]
	AT4G30080.1	ARF16		
	AT1G77850.1	ARF17		
ath-miR169f-3p	AT3G59770.1	SAC9	Probable phosphoinositide phosphatase	[51]
ath-miR394a	AT5G36170.2	HCF109	Proper translation, stability and processing of polycistronic transcripts in chloroplasts	[52]
ath-miR8175	AT2G36250.2	FtsZ2-1	Required for plastid division	[53]

**ARF**: Auxin Response Factor. **SAC9**: Probable phosphoinositide phosphatase. **HCF**: High chlorophyll fluorescent.

## Supplementary Materials

Supplementary materials can be found at https://www.mdpi.com/1422-0067/20/20/5131/s1.
