# Supplementary material for: Exposure to High-Intensity Light Systemically Induces Micro-Transcriptomic Changes in Arabidopsis thaliana Roots
Source: Int J Mol Sci. 2019 Oct 16;20(20):5131. doi: 10.3390/ijms20205131 (PMC6829545; doi:10.3390/ijms20205131)
Supplement: Supplementary file 1 [file ijms-20-05131-s001.pdf]

SUPPLEMENTARY MATERIAL

Supplementary Figure S1. Microtranscriptome analysis pipeline.

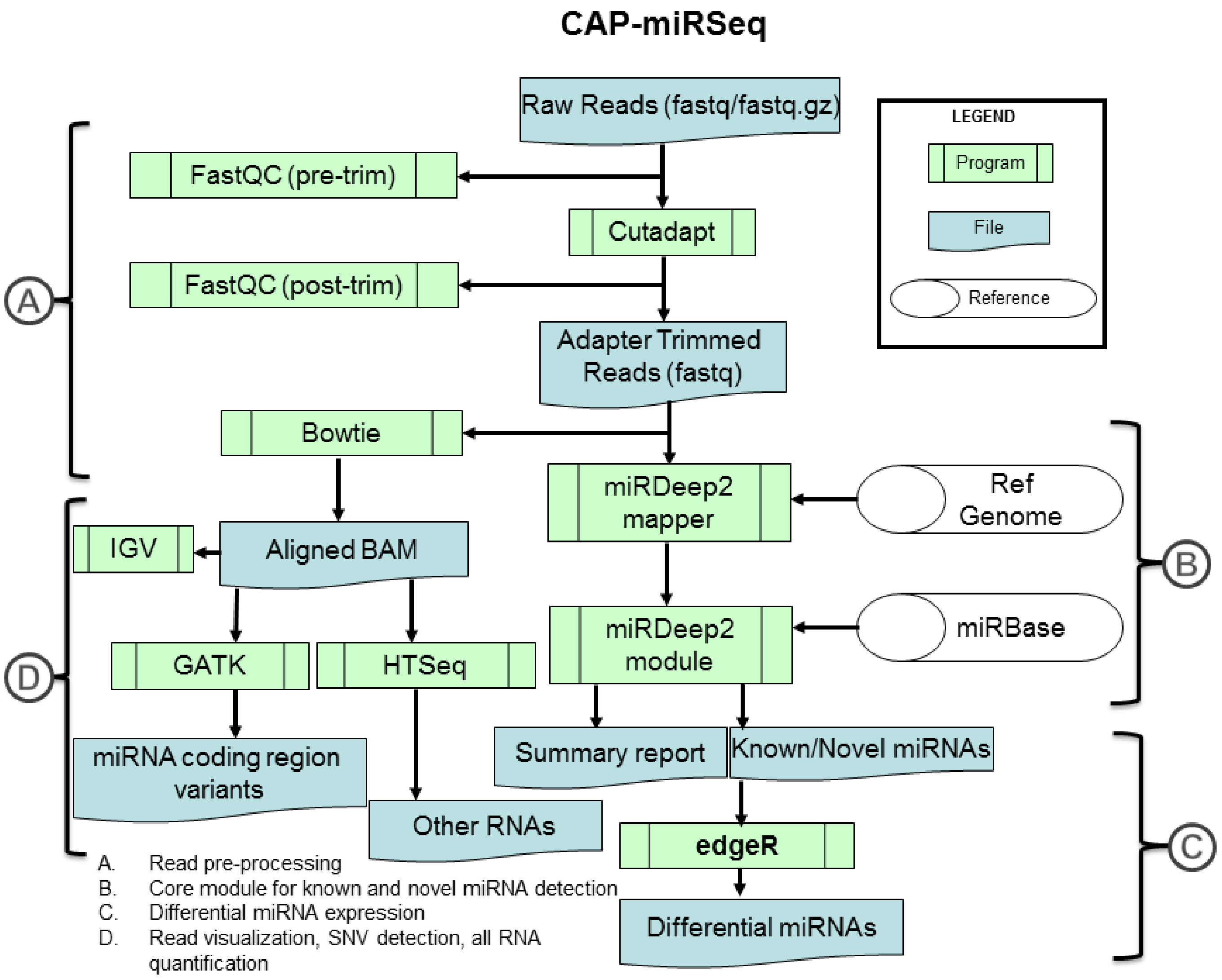

Supplementary Table S1. Significantly changed micro-RNAs.

Id	C_root1	C_root2	0H_Root1	0H_Root2	norm.C_root1	norm.C_root2	norm.0H_Root1	norm.0H_Root2	baseMean	C	0H	FC	log2FoldChange	pvalue	padj	tagwise.dispersion	trended.dispersion
ath-miR156g	37	21	5	3	38	22	6	2	17,17	30	4	0,132	-2,923	2,15E-07	8,77E-06	0,0514	0,0916
ath-miR159c	1926	1416	973	1051	1988	1500	1124	861	1368,06	1744	992	0,568	-0,815	0,000499	0,011615	0,0252	0,0229
ath-miR169f-3p	443	460	253	352	457	487	292	288	381,24	472	290	0,614	-0,703	0,001487	0,027815	0,0206	0,0273
ath-miR160c-3p	129	86	51	65	133	91	59	53	84,09	112	56	0,499	-1,003	0,003513	0,044049	0,0431	0,0424
Id	C_root1	C_root2	0H_Root1	0H_Root2	norm.C_root1	norm.C_root2	norm.0H_Root1	norm.0H_Root2	baseMean	C	0H	FC	log2FoldChange	pvalue	padj	tagwise.dispersion	trended.dispersion
ath-miR319b	193	104	487	583	199	110	562	477	337,28	154	520	3,354	1,746	8,48E-10	1,38E-07	0,0331	0,0279
ath-miR394b-5p	8	6	52	51	8	6	60	42	29,1	7	51	6,799	2,765	1,27E-08	1,03E-06	0,0502	0,0687
ath-miR394a	8	7	51	51	8	7	59	42	29,08	8	50	6,28	2,651	2,66E-08	1,45E-06	0,0485	0,0687
ath-miR158a-3p	17087	7852	25441	29845	17636	8319	29377	24438	19942,39	12978	26908	2,073	1,052	0,000246	0,006692	0,0387	0,0206
ath-miR319a	467	370	696	896	482	392	804	734	602,84	437	769	1,758	0,814	0,000246	0,006692	0,0216	0,0252
ath-miR161.2	111	97	215	214	115	103	248	175	160,21	109	212	1,941	0,957	0,001536	0,027815	0,0363	0,0338
ath-miR165a-3p	3169	1832	3904	4883	3271	1941	4508	3998	3429,52	2606	4253	1,632	0,707	0,002504	0,040807	0,0257	0,0216
ath-miR160b	32	12	62	62	33	13	72	51	42,03	23	62	2,647	1,404	0,003335	0,044049	0,0793	0,0574
ath-miR165b	3581	2106	4348	5452	3696	2231	5021	4464	3853,05	2964	4742	1,6	0,678	0,00344	0,044049	0,0253	0,0215
ath-miR167b	104	115	143	322	107	122	165	264	164,49	114	214	1,878	0,909	0,003964	0,046149	0,0407	0,0335
ath-miR167a-5p	104	115	142	318	107	122	164	260	163,38	114	212	1,858	0,894	0,004582	0,04668	0,0406	0,0336
ath-miR396a-5p	676	493	856	1116	698	522	988	914	780,57	610	951	1,558	0,64	0,004514	0,04668	0,0229	0,0243
Id	C_root1	C_root2	4H_Root1	4H_Root2	norm.C_root1	norm.C_root2	norm.4H_Root1	norm.4H_Root2	baseMean	C	4H	FC	log2FoldChange	pvalue	padj	tagwise.dispersion	trended.dispersion
ath-miR156g	37	21	7	6	34	20	8	7	16,91	27	8	0,267	-1,905	8E-05	0,001681	0,035	0,0256

ath-miR169f-3p	443	460	179	228	405	431	194	249	319,73	418	222	0,529	-0,918	9,28E-05	0,001732	0,0227	0,0256
ath-miR160c-3p	129	86	39	52	118	81	42	57	74,39	100	50	0,499	-1,004	0,002003	0,030597	0,0353	0,0256
ath-miR159c	1926	1416	893	944	1762	1327	968	1029	1271,48	1544	998	0,647	-0,629	0,00311	0,043541	0,0208	0,0256
ath-miR399c-3p	44	38	19	14	40	36	21	15	27,93	38	18	0,474	-1,076	0,003387	0,04377	0,0252	0,0256
Id	C_root1	C_root2	4H_Root1	4H_Root2	norm.C_root1	norm.C_root2	norm.4H_Root1	norm.4H_Root2	baseMean	C	4H	FC	log2FoldChange	pvalue	padj	tagwise.dispersion	trended.dispersion
ath-miR319b	193	104	373	413	177	97	404	450	282,15	137	427	3,115	1,639	2,21E-08	3,71E-06	0,0353	0,0256
ath-miR8175	1	1	10	16	1	1	11	17	7,53	1	14	13,569	3,762	2,02E-07	1,7E-05	0,0088	0,0256
ath-miR157a-3p	65	44	126	119	59	41	137	130	91,75	50	134	2,638	1,4	5,24E-07	2,2E-05	0,0243	0,0256
ath-miR157b-3p	65	44	126	119	59	41	137	130	91,75	50	134	2,638	1,4	4,68E-07	2,2E-05	0,024	0,0256
ath-miR394a	8	7	22	42	7	7	24	46	20,88	7	35	4,943	2,305	6,56E-06	0,000197	0,0567	0,0256
ath-miR394b-5p	8	6	21	42	7	6	23	46	20,37	6	34	5,209	2,381	7,03E-06	0,000197	0,0622	0,0256
ath-miR161.2	111	97	157	181	102	91	170	197	139,99	96	184	1,908	0,932	6,57E-05	0,001576	0,0184	0,0256
ath-miR158a-3p	17087	7852	20193	23756	15628	7359	21896	25892	17693,98	11494	23894	2,079	1,056	0,000347	0,00583	0,0409	0,0256
ath-miR160b	32	12	49	37	29	11	53	40	33,49	20	46	2,293	1,197	0,004398	0,047429	0,0518	0,0256
ath-miR1888a	5	6	15	10	5	6	16	11	9,34	6	14	2,629	1,394	0,004568	0,047429	0,0004	0,0256
ath-miR319a	467	370	498	578	427	347	540	630	485,97	387	585	1,511	0,596	0,004799	0,047429	0,0192	0,0256
ath-miR3932b-5p	0	17	29	30	0	16	31	33	20,02	8	32	3,999	2	0,004149	0,047429	0,1607	0,0256

Supplementary Table S2 . Primer sequences used in the experiments.

**Primer name**	**Accession**	**Primer sequence 5'-3'**	**Application**
PP2AA3_F	**AT1G13320**	TAACGTGGCCAAAATGATGC	RT-qPCR - reference
PP2AA3_R		GTTCTCCACAACCGCTTGGT	RT-qPCR - reference
UPL7_F	**AT3G53090**	TTCAAATACTTGCAGCCAACCTT	RT-qPCR - reference
UPL7_R		CCCAAAGAGAGGTATCACAAGAGACT	RT-qPCR - reference
APX2_F	**AT3G09640**	TCATCCTGGTAGACTGGACAAA	RT-qPCR
APX2_R		CACATCTCTTAGATGATCCACACC	RT-qPCR
CAT2_F	**AT4G35090**	TCTGGTGCTCCTGTATGGAA	RT-qPCR
CAT2_R		TGGTAATCCTCAAGAAGGATAGGA	RT-qPCR
RRTF1_F	**AT4G34410**	TCGGGTATGCATTATCCTAACA	RT-qPCR
RRTF1_R		AAGCTCTTGCTCCGGTGA	RT-qPCR
ELIP1_F	**AT3G22840**	GCACAAAGTTTAGCGACTTGC	RT-qPCR
ELIP1_R		CGCAACGAATCCAACCAT	RT-qPCR
ath-sno85_R1	**AJ505658**	GGTCCAGTTTTTTTTTTTTTTTACATGTA	RT-qPCR - reference
ath-sno85_F1		GGTGCATTCAAAAGCCCTT	RT-qPCR - reference
ath-sno101_R1	**AJ505631**	GACCAGTAGTTATCAACAAGCGA	RT-qPCR - reference
ath-sno101_F1		ACACTTGATCTCTGAACTTCACA	RT-qPCR - reference
ath-sno85_RV1	**AJ505658**	GTGCATTCAAAAGCCCTTACA	TT-RTqPCR - reference
ath-sno85_FW1		GCTTTGAAAGAGAGAGAGAGAG	TT-RTqPCR - reference
ath-sno101_RV1	**AJ505631**	GTTGATAACTACTGGTCTGCTGAT	TT-RTqPCR - reference
ath-sno101_FW1		TGTGAAGAGAGAGAGAGAGAG	TT-RTqPCR - reference
ath-miR157a_RV1	**AT1G66783**	CGCTCTCTAGCCTTCTGTC	TT-RTqPCR
ath-miR157a_FW1		GCGCTAGATTTCCTCCTTGAG	TT-RTqPCR
ath-miR158a-3p_Rv1	**AT3G10745**	GCGTCCCAAATGTAGACAAA	TT-RTqPCR
ath-miR158a-3p_FW1		TTTGGGAGGAGAGAGAGAGAG	TT-RTqPCR
ath-miR158b_RV1	**AT1G55591**	CGCCCCAAATGTAGACAAA	TT-RTqPCR
ath-miR158b_FW1		TTTGGGGATTCGTGGCTGAG	TT-RTqPCR
ath-miR160b-5p_RV1	**AT4G17788**	TGCCTGGCTCCCTGTATG	TT-RTqPCR
ath-miR160b-5p_FW1		CCAGGCACACACACACAGAC	TT-RTqPCR
ath-miR167b-5p_F1	**AT3G63375**	GAAGCTGCCAGCATGA	RT-qPCR
ath-miR167b-5p_R2		CAGGTCCAGTTTTTTTTTTTTTTTAGA	RT-qPCR
ath-miR169f-3p_F1	**AT3G14385**	GCAAGTTGACCTTGGCT	RT-qPCR
ath-miR169f-3p_R1		TCCAGTTTTTTTTTTTTTTTGCAGA	RT-qPCR
ath-miR319b_RV1	**AT5G41663**	TTGGACTGAAGGGAGCTC	TT-RTqPCR
ath-miR319b_FW1		GTCAGTCAAGCAGAGAAGAG	TT-RTqPCR
ath-miR394a_RV1	**AT1G20375**	GTTGGCATTCTGTCCACC	TT-RTqPCR
ath-miR394a_FW1		ATGCCAAAGAGAGAGAGAGAG	TT-RTqPCR
ath-miR8175_1_RV1	**AT2G05455**	GATCCCCGGCAACGGC	TT-RTqPCR
ath-miR8175_1_FW1		GGGGATCAGAGATAGAAAGAG	TT-RTqPCR
RT-ath-snoR85_1	**AJ505658**	TTTGAAAGAGAGAGAGAGAGCTAGAGAACCTAGCTCAATAGGAAGACATGT	RT reaction TT- reference
RT-ath-snoR101_1	**AJ505631**	TGTGAAGAGAGAGAGAGAGAGCTAGAGAACCTAGCTCATTAGGAAGAGCATC	RT reaction TT- reference
RT-ath-miR157a_1	**AT1G66783**	GCTAGATTTCCTCCTTGAGCTAGGTTGACTAGCTCTTCTATATTGATGAC	RT reaction TT
RT-ath-miR158a-3p_1	**AT3G10745**	TTTGGGAGGAGAGAGAGAGAGCTAGAGAACCTAGCTCAGTTGGAGGTGCTTT	RT reaction TT
RT-ath-miR158b_1	**AT1G55591**	TTTGGGGATTCGTGGCTGAGCTAGGTTGACTAGCTCTTCTCTATTTGCTTT	RT reaction TT
RT-ath-miR160b-5p_1	**AT4G17788**	CCAGGCACACACACACAGACGTAGAGAACCTACGTCCACCATACATGGCAT	RT reaction TT
RT-ath-miR319b_1	**AT5G41663**	TCAGTCAAGCAGAGAAGAGCTAGAGAACCTAGCTCAAGACCAATAGGGAG	RT reaction TT
RT-ath-miR394a_1	**AT1G20375**	ATGCCAAAGAGAGAGAGAGAGCTAGAGAACCTAGCTCAATAAGAAGGGAGGT	RT reaction TT
RT-ath-miR8175_1	**AT2G05455**	GGGGATCAGAGATAGAAAGAGCTAGAGAACCTAGCTCAATAGGAAGTGGCGC	RT reaction TT

Supplemetary Table S3. he Hits from the prediction of mRNA targets for confirmed miRNAs, obtained using the psRNATarget software. A higher expectation value (Exp.) indicates less similarity between small RNA and the target candidate (lower probability of true interaction). The target gene regulation can be realized by its cleavage or translation inhibition.

miRNA	miRNA sequence	Exp.	Inhibition	Target Acc.	Target name	Target function
ath-miR160b	UGCCUGGCUCCCUGUAUGCCA	0.5	Cleavage	AT1G77850.1	ARF17	auxin response factor 17
ath-miR160b	UGCCUGGCUCCCUGUAUGCCA	1	Cleavage	AT2G28350.1	ARF10	auxin response factor 10
ath-miR394a	UUGGCAUUCUGUCCACCUCC	1	Cleavage	AT1G27340.1		Galactose oxidase/kelch repeat superfamily protein
ath-miR160b	UGCCUGGCUCCCUGUAUGCCA	1.5	Cleavage	AT4G30080.1	ARF16	auxin response factor 16
ath-miR169f-3p	GCAAGUUGACCUUGGCUCUGC	2.5	Cleavage	AT1G13470.1		Protein of unknown function (DUF1262)
ath-miR169f-3p	GCAAGUUGACCUUGGCUCUGC	2.5	Cleavage	AT5G51220.1		ubiquinol-cytochrome C chaperone family protein
ath-miR394a	UUGGCAUUCUGUCCACCUCC	2.5	Cleavage	AT4G24860.1		P-loop containing nucleoside triphosphate hydrolases superfamily protein
ath-miR394a	UUGGCAUUCUGUCCACCUCC	2.5	Cleavage	AT3G58570.1		P-loop containing nucleoside triphosphate hydrolases superfamily protein
ath-miR169f-3p	GCAAGUUGACCUUGGCUCUGC	3	Cleavage	AT3G59770.1	SAC9	sacI homology domain-containing protein / WW domain-containing protein
ath-miR394a	UUGGCAUUCUGUCCACCUCC	3	Cleavage	AT4G20430.2		Subtilase family protein
ath-miR394a	UUGGCAUUCUGUCCACCUCC	3	Cleavage	AT5G36170.2	HCF109, ATPRFB	high chlorophyll fluorescent 109
ath-miR169f-3p	GCAAGUUGACCUUGGCUCUGC	3.5	Cleavage	AT4G34580.1	COW1, SRH1	Sec14p-like phosphatidylinositol transfer family protein
ath-miR169f-3p	GCAAGUUGACCUUGGCUCUGC	3.5	Cleavage	AT1G67580.2		Protein kinase superfamily protein
ath-miR169f-3p	GCAAGUUGACCUUGGCUCUGC	3.5	Cleavage	AT1G79075.2		other RNA
ath-miR169f-3p	GCAAGUUGACCUUGGCUCUGC	3.5	Cleavage	AT1G42070.1		transposable element gene
ath-miR169f-3p	GCAAGUUGACCUUGGCUCUGC	3.5	Cleavage	AT3G16760.1		Tetratricopeptide repeat (TPR)-like superfamily protein
ath-miR169f-3p	GCAAGUUGACCUUGGCUCUGC	3.5	Translation	AT5G55780.1		Cysteine/Histidine-rich C1 domain family protein
ath-miR169f-3p	GCAAGUUGACCUUGGCUCUGC	3.5	Cleavage	AT5G18830.3	SPL7	squamosa promoter binding protein-like 7
ath-miR394a	UUGGCAUUCUGUCCACCUCC	3.5	Translation	AT5G20580.1		BEST Arabidopsis thaliana protein match is
ath-miR394a	UUGGCAUUCUGUCCACCUCC	3.5	Translation	AT1G01320.2		Tetratricopeptide repeat (TPR)-like superfamily protein
ath-miR394a	UUGGCAUUCUGUCCACCUCC	3.5	Cleavage	AT3G48460.1		GDSL-like Lipase/Acylhydrolase superfamily protein
ath-miR394a	UUGGCAUUCUGUCCACCUCC	3.5	Cleavage	AT2G33585.1		unknown protein; has 31 Blast hits to 31 proteins in 12 species
ath-miR394a	UUGGCAUUCUGUCCACCUCC	3.5	Cleavage	AT5G56630.1	PFK7	phosphofructokinase 7
ath-miR394a	UUGGCAUUCUGUCCACCUCC	3.5	Cleavage	AT1G40104.1		unknown protein; FUNCTIONS IN
ath-miR394a	UUGGCAUUCUGUCCACCUCC	3.5	Translation	AT5G26749.2		C2H2 and C2HC zinc fingers superfamily protein

ath-miR394a	UUGGCAUUCUGUCCACCUCC	3.5	Cleavage	AT5G19097.1		transposable element gene
ath-miR394a	UUGGCAUUCUGUCCACCUCC	3.5	Cleavage	AT5G63120.2		P-loop containing nucleoside triphosphate hydrolases superfamily protein
ath-miR394a	UUGGCAUUCUGUCCACCUCC	3.5	Cleavage	AT3G23300.1		S-adenosyl-L-methionine-dependent methyltransferases superfamily protein
ath-miR8175	GAUCCCCGGCAACGGCGCCA	3.5	Cleavage	AT5G28641.1		transposable element gene
ath-miR8175	GAUCCCCGGCAACGGCGCCA	3.5	Cleavage	AT1G22040.1		Galactose oxidase/kelch repeat superfamily protein
ath-miR8175	GAUCCCCGGCAACGGCGCCA	3.5	Cleavage	AT5G47500.1		Pectin lyase-like superfamily protein
ath-miR8175	GAUCCCCGGCAACGGCGCCA	3.5	Cleavage	AT3G05520.2		Subunits of heterodimeric actin filament capping protein Capz superfamily
ath-miR8175	GAUCCCCGGCAACGGCGCCA	3.5	Cleavage	AT5G36890.2	BGLU42	beta glucosidase 42
ath-miR8175	GAUCCCCGGCAACGGCGCCA	3.5	Translation	AT5G55340.1		MBOAT (membrane bound O-acyl transferase) family protein
ath-miR160b	UGCCUGGCUCCCUGUAUGCCA	4	Cleavage	AT1G07970.1		CONTAINS InterPro DOMAIN/s
ath-miR160b	UGCCUGGCUCCCUGUAUGCCA	5	Cleavage	ATMG00880.1	ORF187	hypothetical protein
ath-miR160b	UGCCUGGCUCCCUGUAUGCCA	5	Translation	AT4G19640.1	ARA7, ARA-7, ATRABF2B	Ras-related small GTP-binding family protein
ath-miR160b	UGCCUGGCUCCCUGUAUGCCA	5	Translation	AT1G32270.1	ATSYP24, SYP24	syntaxin, putative
ath-miR160b	UGCCUGGCUCCCUGUAUGCCA	5	Cleavage	AT1G64190.1		6-phosphogluconate dehydrogenase family protein
ath-miR160b	UGCCUGGCUCCCUGUAUGCCA	5	Cleavage	AT3G09450.1		CONTAINS InterPro DOMAIN/s
ath-miR160b	UGCCUGGCUCCCUGUAUGCCA	5	Cleavage	AT1G79830.1	GC5	golgin candidate 5
ath-miR160b	UGCCUGGCUCCCUGUAUGCCA	5	Cleavage	AT2G07682.1		transposable element gene
ath-miR160b	UGCCUGGCUCCCUGUAUGCCA	5	Cleavage	AT4G15100.1	scpl30	serine carboxypeptidase-like 30
ath-miR8175	GAUCCCCGGCAACGGCGCCA	5	Cleavage	AT3G22850.1		Aluminium induced protein with YGL and LRDR motifs
ath-miR8175	GAUCCCCGGCAACGGCGCCA	5	Cleavage	AT5G53440.1		unknown protein; FUNCTIONS IN
ath-miR8175	GAUCCCCGGCAACGGCGCCA	5	Cleavage	AT4G23440.1		Disease resistance protein (TIR-NBS class)
ath-miR8175	GAUCCCCGGCAACGGCGCCA	5	Translation	AT4G33430.1	BAK1,RKS10, SERK3	BRI1-associated receptor kinase
ath-miR8175	GAUCCCCGGCAACGGCGCCA	5	Cleavage	AT3G25560.3	NIK2	NSP-interacting kinase 2
ath-miR8175	GAUCCCCGGCAACGGCGCCA	5	Cleavage	AT2G36250.2	FTSZ2-1, ATFTSZ2-1	Tubulin/FtsZ family protein
ath-miR8175	GAUCCCCGGCAACGGCGCCA	5	Cleavage	AT3G25560.2	NIK2	NSP-interacting kinase 2
ath-miR8175	GAUCCCCGGCAACGGCGCCA	5	Cleavage	AT3G47990.1	SIS3	SUGAR-INSENSITIVE 3
ath-miR8175	GAUCCCCGGCAACGGCGCCA	5	Cleavage	AT1G67320.1		DNA primase, large subunit family
